# Protect Effects of Seafood-Derived Plasmalogens Against Amyloid-Beta (1–42) Induced Toxicity via Modulating the Transcripts Related to Endocytosis, Autophagy, Apoptosis, Neurotransmitter Release and Synaptic Transmission in SH-SY5Y Cells

**DOI:** 10.3389/fnagi.2021.773713

**Published:** 2021-11-26

**Authors:** Junli Feng, Gongshuai Song, Qing Shen, Xi Chen, Qingcheng Wang, Shunyuan Guo, Manman Zhang

**Affiliations:** ^1^Zhejiang Province Joint Key Laboratory of Aquatic Products Processing, Collaborative Innovation Center of Seafood Deep Processing, Institute of Seafood, Zhejiang Gongshang University, Hangzhou, China; ^2^Zhejiang Provincial People’s Hospital, Hangzhou, China; ^3^Department of Cardiology, Hangzhou Linping Hospital of Traditional Chinese Medicine, Hangzhou, China; ^4^Department of Neurology, The First Affiliated Hospital of Wenzhou Medical University, Wenzhou, China

**Keywords:** Alzheimer’s disease, plasmalogens, SH-SY5Y cells, gene expression, transcriptional profiles

## Abstract

To investigate the underlying mechanisms of decreased plasmalogens (Pls) levels in neurodegenerative diseases, here the effects of seafood-derived Pls on undifferentiated and differentiated human SH-SY5Y neuroblastoma cells exposed to amyloid-β_1–42_ was analyzed. Transcriptional profiles indicated that a total of 6,581 differentially expressed genes (DEGs) were significantly identified among different experimental groups, and KEGG analysis indicated that these DEGs were related to AD, endocytosis, synaptic vesicle cycle, autophagy and cellular apoptosis. After Pls treatment, the striking expression changes of *ADORA2A*, *ATP6V1C2*, *CELF6*, and *SLC18A2* mRNA strongly suggest that Pls exerts a beneficial role in alleviating AD pathology partly by modulating the neurotransmitter release and synaptic transmission at the transcriptional level. Besides these, GPCRs are also broadly involved in Pls-signaling in neuronal cells. These results provide evidence for supporting the potential use of Pls as an effective therapeutic approach for AD.

## Introduction

Alzheimer’s disease (AD), a progressive neurodegenerative disorder, often occurs among aging people (Chung et al., 2019). This disease is characterized by memory loss and cognitive impairment. The pathological features of AD are the deposition of amyloid-β (Aβ) plaques and formation of neurofibrillary tangles, which is primarily constituted by hyperphosphorylated tau proteins in extra- and intra-nerve cells, leading to synaptic dysfunction and neuronal death. Although different therapeutic approaches have been proposed, only few are tested effectively to block AD progression (Sorrentino et al., 2017). In addition to aging, studies have shown that several risk factors play a crucial role in predisposing for AD. For example, smoking, midlife obesity, hypertension, type 2 diabetes, hypercholesterolemia and history of traumatic brain injury are strongly associated with the onset of AD (Meco et al., 2020).

The molecular pathogenesis of this age-related cognitive decline remains incompletely defined (Wan et al., 2020). Intraneuronal Aβ accumulation is believed to trigger neurodegeneration by disrupting synaptic transmission and endosomal, lysosomal/proteasomal, and mitochondrial functions as well as by facilitating the hyperphosphorylation of microtubular tau protein (Omtri et al., 2018). Genome-wide association studies on AD patient brains have demonstrated significant expression changes in genes that regulate vesicular trafficking, cytoskeleton, energy metabolism, inflammation, ubiquitin-proteosome system, and autophagy (Liang et al., 2007). Besides these, it has been reported that the phosphoinositide 3-kinase (PI3K)/AKT-mTOR signaling pathway, nuclear factor kappa B (NF-κB), and mitogen-activated protein kinase (MAPK) pathways are activated in neurons in several neuropathological conditions (Zheng et al., 2017; Coelho et al., 2019; Saha et al., 2020).

Plasmalogens (Pls) are a special class of dietary glycerophospholipids which can be easily found in animal foodstuffs such as poultry, livestock and seafood. Pls are abundant in the nervous system, especially in the white matter, where Pls are localized in the cytoplasmic side of myelin (Che et al., 2018). Besides their contribution to membrane integrity as structural components, Pls are also involved in multiple cellular processes such as membrane fusion, ion transport, cholesterol efflux, and generation of secondary messengers (Su et al., 2019). Several literatures have reported a direct link between Pls deficiency and AD pathology, whereas the administration of Pls (1 mg/day) was effective to improve cognitive function of mild AD patients (Ginsberg et al., 1995; Wood et al., 2011; Fujino et al., 2017). Che et al. (2018) have reported Pls (20 μg/mL in the medium) could significantly decrease intracellular and extracellular Aβ_42_ levels of CHO-APP/PS1 cells, whereas another study suggested that Pls (5 and 20 μg/mL in the medium) prevented neuronal cell death by activating G-protein coupled receptors (GPCRs) to induce ERK and Akt cellular signaling (Hossain et al., 2013, 2016). However, limited genes have been analyzed in these studies. Therefore, it is necessary to explore the role of Pls in the pathogenesis of AD as a whole.

Aβ peptide is a product of its precursor amyloid polypeptide protein by sequential proteolytic cleavages, and it has various conformations. Among them, Aβ_1–42_ easily forms insoluble aggregates, which are the predominant fibers found in senile neuritic plaques of AD brains (Selkoe, 2001). Previous studies proved that Aβ_1–42_ has neurotoxic potential, interfering with synaptic plasticity and affecting several cellular signaling pathways (Selkoe, 2001). Thus, neurons exposed to appropriate Aβ_1–42_ (less than 10 μM) are routinely used to obtain the *in vitro* AD models (Arbo et al., 2017; Coelho et al., 2019).

In this study, SH-SY5Y neuroblastoma cells were used to obtain human neuron-like cells using the sequential *all-trans* retinoic acid (ATRA) differentiation and brain-derived neurotrophic factor (BDNF) maturation program (Encinas et al., 2000; Goldie et al., 2014). Then, *in vitro* AD model cells were constructed through Aβ_1–42_ induction, and they were treated with seafood-derived Pls (10 μg/mL) for 24 h. Finally, the transcriptional profiles of AD model cells before and after Pls treatment were characterized, and the potential effects of seafood-derived Pls on the AD-related pathological process were uncovered. Results of this study may help us to fully understand the beneficial effects of seafood-derived Pls on pathology of patients with AD.

## Materials and Methods

### Cell Culture and Treatment

The SH-SY5Y human neuroblastoma cell line obtained from BeNa Culture Collection (Beijing, China). The SH-SY5Y cells were grown for three generations before experiments, and they were used in a low passage number (<13). Cells were cultured in flasks in a humidified incubator at 37°C and 5% CO_2_ (MCO-15AC, Sanyo Electric Co., Osaka, Japan), and the culture medium was replaced thrice per week. The SH-SY5Y cells were cultured in Dulbecco′s Modified Essential Medium (DMEM, Gibco, San Francisco, CA, United States) supplemented with 10% heat-inactivated fetal bovine serum (FBS, Gibco) and 100 U/mL penicillin/streptomycin (Gibco), and were split into three groups at 80% confluence.

For the control group, the cells were still cultured in DMEM containing 10% FBS and 100 U/mL penicillin/streptomycin to reach 80% confluence, and were harvested at this stage (at day 3). For the other experiment groups, ATRA (Sigma-Aldrich, St. Louis, MO, United States) was added in the medium (DMEM + 10% FBS + 10 μM ATRA + 100 U/mL penicillin/streptomycin), and the same culture medium was changed on day 3. On day 6, the culture medium was changed to serum-free BDNF (Life Technologies, Carlsbad, CA, United States) medium (DMEM + 50 ng/mL BDNF + 100 U/mL penicillin/streptomycin). After 7 days of BDNF exposure, neuron-like phenotype were obtained on day 13. To obtain cells of Pls group (Pls), these neuron-like cells were further treated with seafood-derived Pls (10 μg/mL in medium) for 24 h. For the AD model group (AD), the neuron-like cells were treated with Aβ_1–42_ (1.0–4.0 μM, Sigma-Aldrich) for 24 h before cells collection, whereas cells in the AD model + Pls group (AD_Pls) were further treated with 10 μg/mL seafood-derived Pls in medium for another 24 h and harvested at day 15.

The seafood-derived Pls was extracted and purified from Mussel (*Mytilus edulis*) in the laboratory, according to the method described in a previous study (Wang et al., 2021). The purity of the obtained Pls was 91.56%, with the major components of phosphatidylethanolamine plasmalogens (50.13%) and phosphatidylcholine plasmalogens (41.43%) when analyzed by HPLC-ELSD (Wang et al., 2021). The remaining 8.44% contained 3.12% PC and 5.08% PE. The proportions of the unsaturated fatty acid in Pls and phospholipids were 63.07 and 58.43%. eicosapentaenoic acid was the major constituent of unsaturated fatty acids, which accounted for 45.82 and 42.35% in Pls and phospholipids, respectively (Song et al., 2020; Wang et al., 2021; Zhang et al., 2021). During the culture and differentiation stages, SH-SY5Y cells were visualized by a Zeiss Axiovert 200 inverted fluorescence microscope (Carl Zeiss, Oberkochen, Germany), and documented with AxioVisionLE (Carl Zeiss).

### The 4′,6-Diamidino-2-Phenylindole and Propidium Iodide (PI) Staining

The death of SH-SY5Y cells treated with different concentrations of Aβ_1–42_ was measured by 4′,6-Diamidino-2-Phenylindole (DAPI) (Sigma-Aldrich) and Propidium Iodide (PI) (Sigma-Aldrich) staining. Briefly, differentiated SH-SY5Y cells were cultured on coverslips in a 24-well plate. After the cells adhered, 5 mg/L DAPI was added to the culture supernatant and cultured overnight in dark. Then, Aβ_1–42_ was added to each well to the final concentrations of 1, 2, and 4 μM. After treatment with Aβ_1–42_ for 24 h, 50 mg/L PI was added to each well and stained for 3–5 min. Then, cells were observed under a fluorescence microscope (Carl Zeiss) and analyzed by AxioVisionLE software. Five images were obtained for each cell sample. The total number of nuclei was assessed by DAPI staining, while the nuclei of dead cells were determined by PI staining. Therefore, the cell death rate was determined by comparing the number of PI positive cells to the number of total cells in the images.

### RNA Extraction and RNA-Seq Library Preparation

Total RNA was extracted from SH-SY5Y cells of different groups using TRIzol Reagent (Invitrogen, CA, United States). The RNA quantity and quality were analyzed using a NanoDrop ND-1000 instrument (Agilent, CA, United States). The mRNA was purified from 5 μg of total RNA using poly-T oligo-attached magnetic beads after two rounds of purification. The obtained mRNA was fragmented using divalent cations under elevated temperature, and then reverse-transcribed to construct the cDNA library using the mRNASeq sample preparation kit (Illumina, San Diego, United States). The sequencing was performed on an Illumina Novaseq^TM^ 6000 platform (LC Sciences, Houston, Texas, United States). Three biological repeats were assessed for each sample, and all of the data were expressed as the mean ± standard deviation (SD). The raw datasets of sequencing have been submitted to the NCBI Short Read Archive under the accession code PRJNA728528.

### RNA-Seq Reads Analysis

The raw transcriptome data were first processed by removing adaptor-containing sequences, poly-N, reads shorter than 150 bp, and low-quality reads. Then the valid reads were aligned to the homo-genome (http://www.ensembl.org/Homo_sapiens/Info/Index) using the HISAT package, which allowed multiple alignments (up to 20 by default) and 2 mismatches while mapping the reads to the reference genes. Mapped reads of each sample were assembled using StringTie, and all of the transcriptome data were merged to generate a comprehensive transcriptome using Perl scripts software. StringTie and edgeR were used to calculate the expression levels of all transcripts by calculating FPKM. These mRNAs were further analyzed using GO (gene ontology) enrichment analysis and KEGG (Kyoto Encyclopedia of Genes and Genomes) signaling pathway enrichment analysis.

### Quantitative RT-PCR

To validate the RNA-seq results, the abundance of *ADORA2A*, *APP*, *ATP6V1C2*, *Bcl-2*, *DGKK*, *GSAP*, *GSK3*, *IL33*, *PSEN1*, and *SLC18A2* mRNAs in different experimental groups were analyzed in parallel using an ABI Prism 7500 Sequence Detection System (PE Applied Biosystems, CA, United States). Briefly, total RNA was extracted from samples with three biological repeats, and converted to cDNA using the cDNA reverse transcription kit (Applied Biosystems). Quantitative RT-PCR (qRT-PCR) primers used for determining the transcript abundance of selected mRNAs are listed in Supplementary Table 1. *GAPDH* was used as the housekeeping gene. The qPCR amplification was initiated by denaturation at 95°C for 10 s, followed by 40 cycles of 95°C for 10 s and 60°C for 30 s. Each reaction was repeated three times. The specificity of the amplification was examined by melting curve analysis. The relative fold changes of the tested genes were calculated using the 2^–ΔΔCt^ method.

### Statistics Analysis

All results in this study are represented as the average ± standard deviation (SD) from three independent experiments. Statistical analysis was performed using Microsoft Excel, and one-way analysis of variance (ANOVA) followed by Duncan’s new multiple range tests. Differences were assumed to be statistically significant, and indicated with different letters, for *P* < 0.05.

## Results

### Morphological Characteristics of Cultured SH-SY5Y

**FIGURE 1:**
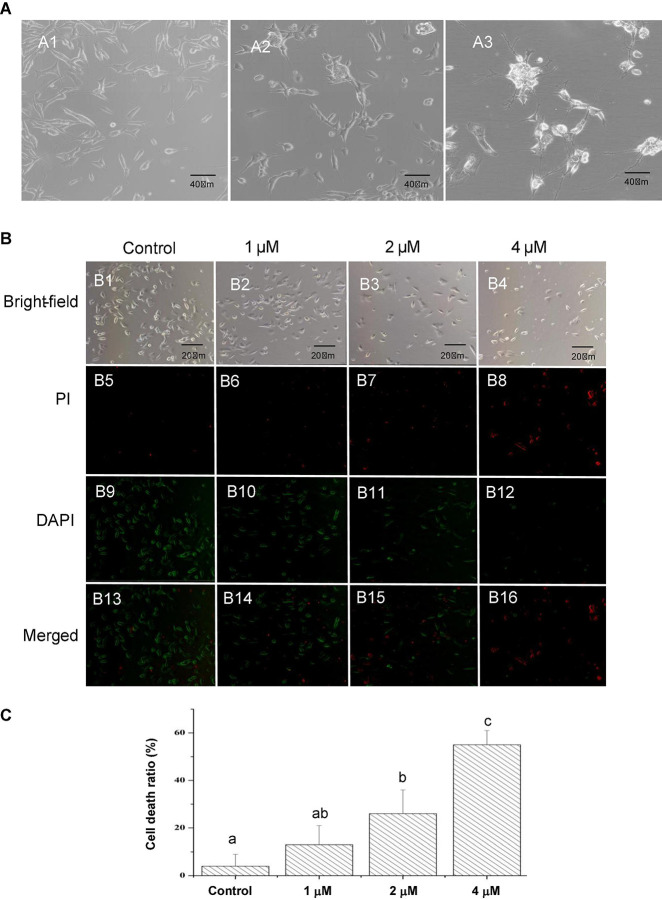
Characteristics of cultured SH-SY5Y cells and effects of Aβ_1–42_ on death of SH-SY5Y cells. **(A)** Morphology of SH-SY5Y cells at different differentiation stages observed using an Axiovert inverted microscope. A1, untreated SH-SY5Y cells (×200 magnification). A2, SH-SY5Y cells treated with ATRA for 5 days (×200 magnification). These cells demonstrated an increased death rate and a more polar morphology, as cell bodies extended longer and networks began to form. A3, after further maturation with BDNF, the cells migrated to clusters and networks became increasingly complex (×200 magnification). **(B)** The SH-SY5Y cells were treated with different concentrations of Aβ_1–42_ for 24 h, the cell death rates were determined by comparing the number of dead cells to the number of total cells in the images (×200 magnification). **(C)** Quantification of cell death ratios after 24 h treatment with the indicated concentrations of Aβ_1–42_. The different letters indicated a significant difference between experiment groups at *p* < 0.05 (Duncan’s new multiple range test).

In the present study, SH-SY5Y cells were imaged at each stage of treatment using the Zeiss Axiovert 200 inverted microscope. As shown in Figure 1A, SH-SY5Y cells treated with ATRA for 5 days had reduced proliferation and presented a more polar morphology, with cell bodies being extended and networks beginning to develop. After further maturation with BDNF, cells migrated to clusters, and the cellular networks became increasingly complex, which were similar to the mature neurons. These mature neuron-like cells were further treated with Pls and Aβ_1–42_ alone or in combination, to obtain cells of Pls group, AD group, and AD_Pls, respectively. However, no obvious morphological differentiation was observed during Pls or Aβ_1–42_ treatments.

### Effects of Aβ_1–42_ on Death of SH-SY5Y Cells

To assess the effects of Aβ_1–42_ on cell death, equal numbers of differentiated SH-SY5Y cells were seeded in each well of a 24-well plate. Nevertheless, the clusters and network structures of the neuron-like SH-SY5Y cells disappeared after trypsin hydrolysis during transferring from the culture flasks to coverslips. Figure 1B presented that the cell death ratio notably increased with an increasing in Aβ_1–42_ concentrations, indicating that the damage of Aβ_1–42_ on SH-SY5Y cells was dose-dependent. Figure 1C represented that treatment with 2 μM of Aβ_1–42_ leads to 26% cell death, while 55% of the SH-SY5Y cells died when treatment with 4 μM of Aβ_1–42_ for 24 h. To ensure the optimal induction result and an appropriate cell concentration, 2 μM of Aβ_1–42_ was finally used in subsequent experiments.

### Transcriptome Sequencing of SH-SY5Y

To investigate the effect of Pls on AD development, the transcriptomes of samples from normal SH-SY5Y cells, AD model and AD_Pls SH-SY5Y cells were sequenced. The RNA sequence results are summarized in Table 1. A total of 466,789,804 raw reads were obtained, of which the valid reads was 428,666,272. Further analysis of these high-quality cleaned reads generated 208,460 assembled transcripts, which corresponded to 58,826 expression genes. In the alignment analysis, the ratio of valid reads which were mapped to homo-genome came up to 96.40–96.72%. The Q30 values were greater than 97.43%, and the GC contents were ranged from 51 to 53%. Besides these, the Pearson correlation coefficients of these transcriptome profiles among libraries and biological repeats further proved the RNA sequence data were reliable (Supplementary Figure 1).

**TABLE 1:** Summary of the transcriptome sequencing database.

Group	Total reads	Valid reads	Mapped reads	Unique mapped reads	Multiple mapped reads	GC%	≥ Q30%
**ADmodel**	160,677,104	147,325,300	142,448,010 (96.69%)	114,367,872 (77.62%)	28,080,138 (19.06%)	51.50	97.51
**AD model + Pls**	147,231,378	137,964,148	133,011,556 (96.40%)	105,793,995 (76.68%)	27,217,561 (19.72%)	51.00	97.43
**Control**	158,881,322	143,376,824	138,685,824 (96.72%)	107,595,429 (75.02%)	31,090,395 (21.70%)	53.00	97.51
**Total**	466,789,804	428,666,272	414,145,390	–	–	–	–

### Transcriptional Profiles of Different Experimental Groups

**FIGURE 2:**
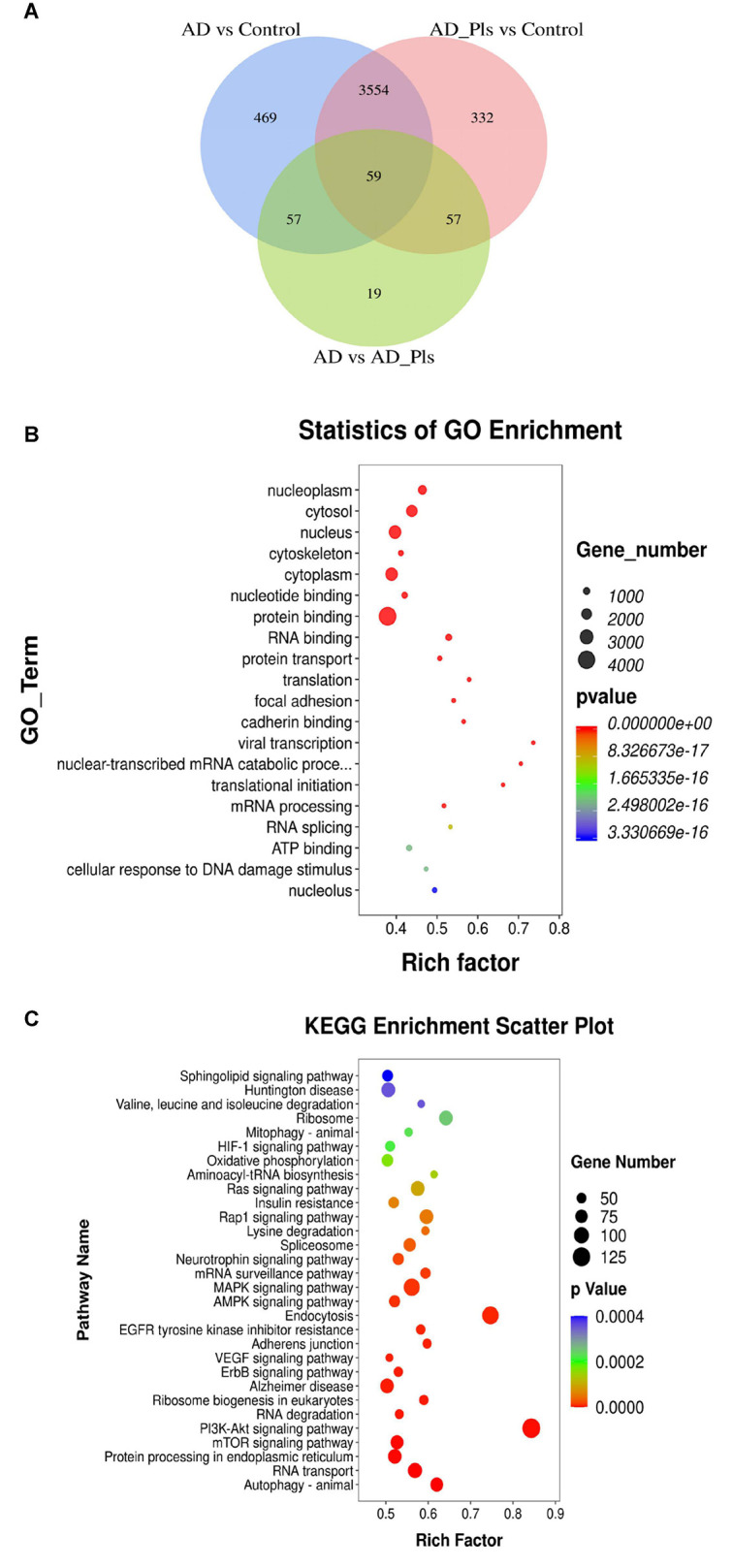
Numbers of DEGs between experimental groups, and their corresponding GO and KEGG enrichment data. **(A)** Venn diagram showing the DEGs significantly identified by comparison of AD model and control groups, AD model + Pls and control groups, and AD model and model + Pls groups (fold change > 2 or fold change < 0.5). **(B)** GO function enrichment analysis of DEGs. **(C)** KEGG pathway enrichment analysis of DEGs.

As shown in Figure 2, 6,581 differentially expressed genes (DEGs) were identified as significant when the experimental groups were compared with each other. Using a fold change cutoff ratio of ≥ 2 or ≤ 0.5, 4,139 DEGs (3721 up-regulated, 418 down-regulated) were further identified when the AD model was compared with control cells. The comparison of AD_Pls and control groups yielded 4,002 DEGs (3578 up-regulated, 424 down-regulated), whereas the comparison of AD and AD_Pls groups yielded 192 DEGs (105 up-regulated, 87 down-regulated). And overall, 59 DEGs were common in all of the three comparisons.

Then, the potential biological functions of these DEGs were analyzed. In the GO analysis performed among control, AD and AD_Pls groups, the most significantly enriched GO terms were “protein binding,” “nucleus,” cytoplasm,” “cytosol,” and “nucleoplasm”(Figure 2B). Similarly, KEGG enrichment analysis indicated that “PI3K-Akt signaling pathway,” “Endocytosis,” “MAPK signaling pathway,” “Alzheimer disease,” “mTOR signaling pathway,” “protein processing in endoplasmic reticulum,” and “Autophagy” were the most represented pathways (Figure 2C). Therefore, these pathways were specifically analyzed.

### Differentially Expressed Genes Directly Involved in Alzheimer’s Disease

The 86 DEGs directly involved in “Alzheimer disease” pathway were first analyzed. As results presented in Table 2, the expression of several hallmarks of AD, such as *APAF1*, *APP*, and *PSEN1* were obviously up-regulated in AD model and AD_Pls cells. Compared with AD model cells, the increased levels of these DEGs were less significant in the AD_Pls group. Moreover, the results indicated that more than half of the AD-related DEGs (48 out of 86) were involved in oxidative phosphorylation (OXPHOS). And expression levels of most OXPHOS transcripts were decreased in AD model and AD_Pls groups. Besides these, several DEGs involved in AD-related calcium signaling pathway (such as *CACNA1D* and *ITPR2*), were also significantly altered (Supplementary Table 2).

**TABLE 2:** List of the significant (*p* < 0.05) AD-associated DEGs and respective fold changes identified among different groups.

Gene name	Fold change (AD model/control)	Fold change (AD model + Pls/control)	Description
**Amyloid β formation**			
*APAF1*	3.85	2.72	Apoptotic peptidase activating factor 1
*APP*	3.92	3.73	Amyloid beta precursor protein
*IDE*	3.77	2.80	Insulin degrading enzyme
*MME*	5.22	3.23	Membrane metalloendopeptidase
*PSEN1*	4.73	4.34	Presenilin 1
**AD-related oxidative phosphorylation**			
*ATP5F1E*	0.61	0.70	ATP synthase F1 subunit epsilon, OXPHOS complex V
*COX4I2*	0.24	0.37	Cytochrome c oxidase subunit 4I2, OXPHOS complex IV
*COX7A2L*	0.39	0.43	Cytochrome c oxidase subunit 7A2 like, OXPHOS complex IV
*CYC1*	0.52	0.57	Cytochrome c1, OXPHOS complex III
*NDUFA3*	0.54	0.72	NADH:ubiquinone oxidoreductase subunit A3, OXPHOS complex I
*NDUFS8*	0.57	0.74	NADH:ubiquinone oxidoreductase core subunit S8, OXPHOS complex I
*SDHD*	1.58	1.76	Succinate dehydrogenase complex subunit D, OXPHOS complex II
*UQCRB*	0.48	0.52	Ubiquinol-cytochrome c reductase binding protein, OXPHOS complex III

### Differentially Expressed Genes Involved in PI3K-Akt/mTOR and Mitogen-Activated Protein Kinase Signaling Pathways

Then, 178 DEGs involved in PI3K-AKT/mTOR signaling pathway were identified. As the results shown in Table 3, most of these DEGs were up-regulated in either AD or AD_Pls groups compared with the control cells. However, the up-regulation extents were less significant after Pls treatment, showing by the 102 of 178 down-regulated DEGs involved in PI3K-AKT/mTOR signaling pathway when the AD_Pls group was compared with AD models. Among them, the down-regulation of *SOS1* was most significantly, whereas *ATP6V1C2* was notably up-regulated after Pls treatment.

**TABLE 3:** List of the significant (*p* < 0.05) PI3K-AKT/mTOR and MAPK signaling pathways related DEGs and respective fold changes identified among different groups.

Gene name	Fold change (AD model/control)	Fold change (AD model + Pls/control)	Description
**PI3K-AKT/mTOR signaling pathway**			
*ATP6V1C2*	0.15	2.10	ATPase H^+^ transporting V1 subunit C2
*FN1*	2.02	1.64	Fibronectin 1
*FOXO3*	4.91	3.94	Forkhead box O3
*ITGA9*	3.19	2.27	Integrin subunit alpha 9
*KIT*	4.55	3.37	KIT proto-oncogene receptor tyrosine kinase
*LAMA1*	3.49	2.47	Laminin subunit alpha 1
SOS1*	4.56	1.64	SOS Ras/Rac guanine nucleotide exchange factor 1
**MAPK signaling pathway**			
*MAP3K13*	2.57	1.48	Mitogen-activated protein kinase kinase kinase 13
*NFATC3*	3.47	1.53	Nuclear factor of activated T cells 3

*Indicate genes involved in both PI3K-AKT/mTOR and MAPK signaling pathways.

The activation of MAPK pathway plays a pivotal role in Aβ-induced neuroinflammation (Zaretsky and Zaretskaia, 2021). Consistent with previous study, 110 DEGs that participated in the MAPK pathway were identified as significant, and 84 of 110 DEGs were up-regulated in the AD group compared with controls. After further treatment with Pls, most of these DEGs were down-regulated, especially *SOS1*, *MAP3K13*, and *NFATC3*.

### Differentially Expressed Genes Involved in Endocytosis and Synaptic Vesicle Cycle

Increasing evidences have reported that the onset and progression of AD are associated with endocytosis, as Aβ enters a cell by endocytosis, and then the endocytic vesicle is merged with a lysosome for degradation the peptide (Muraleva et al., 2019). In this study, 115 DEGs related to endocytosis were identified, and most of them were up-regulated in both AD and AD_Pls groups. Further comparison between the AD and AD_Pls groups indicated that the up-regulation of *CBL* and down-regulation of *ARF1* were the most significant after treatment with Pls.

Communication between neurons is mediated by the release of neurotransmitters from the synaptic vesicle, thus impairment of synaptic vesicle dynamics is believed to be one cause of cognitive defects in AD. In this study, 29 DEGs involved in synaptic vesicle cycle were identified. Among them, the calcium sensor *SYT1* was up-regulated by 4.68- and 4.62-folds in AD and AD_Pls groups, respectively (Table 3). The expression levels of *ATP6V1C2* (responsible for the vesicle acidification) and *SLC18A2* (involved in the vesicle retrieved), were notably decreased to 0.03- and 0.0005-fold of control cells in the AD group, whereas their levels recovered to 2.10- and 0.47-fold of controls in AD_Pls group.

### Differentially Expressed Genes Related to Autophagy and Apoptosis

The dramatic increase in autophagic vacuoles is another feature of AD. To explore the changes in autophagy in AD and AD_Pls cells, 81 autophagy-related DEGs were assessed. Table 4 showed that most of these DEGs were up-regulated in AD and AD_Pls groups, but the up-regulation levels of *MTMR3 and STX17* were markedly reduced after Pls treatment. Nevertheless, the transcript levels of genes encoding lysosomal proteolysis (*CTSB* and *CTSD*) did not significantly change among different experimental groups.

**TABLE 4:** List of significant (*p* < 0.05) endocytosis, synaptic vesicle cycle, autophagy, and apoptosis-associated DEGs and respective fold changes identified among different groups.

Gene name	Fold change (AD model/control)	Fold change (AD model + Pls/control)	Description
**Endocytosis**			
*ARF1*	1.22	0.44	ADP ribosylation factor 1
*CBL*	0.35	1.94	Cbl proto-oncogene
*CHMP4A*	1.60	2.45	Charged multivesicular body protein 4A
*SH3KBP1*	1.64	2.28	SH3 domain containing kinase binding protein 1
**Synaptic vesicle cycle**			
*ATP6V1C2*	0.15	2.10	ATPase H + transporting V1 subunit C2
*SLC18A2*	0.00	0.47	Solute carrier family 18 member A2
*SYT1*	4.68	4.62	Synaptotagmin 1
**Autophagy**			
*ATG2B*	4.15	3.76	Autophagy related 2B
*ATG4A*	2.74	2.37	Autophagy related 4A cysteine peptidase
*ITPR1*	4.87	4.25	Inositol 1,4,5-trisphosphate receptor type 1
*LAMP1*	1.74	1.42	Lysosomal associated membrane protein 1
*MTMR3*	5.47	3.79	Myotubularin related protein 3
PIK3R1*	2.10	3.24	Phosphoinositide-3-kinase regulatory subunit 1
*STX17*	3.64	1.35	Syntaxin 17
**Apoptosis**			
*CASP2*	2.33	1.13	Caspase 2
*DDIT3*	1.54	2.75	DNA damage inducible transcript 3
*ITPR2*	5.06	2.92	Inositol 1,4,5-trisphosphate receptor type 2
*PTPN13*	5.34	5.35	Protein tyrosine phosphatase, non-receptor type 13

*indicate genes involved in both autophagy and apoptosis.

Autophagy is closely related to cell apoptosis, thus the apoptosis pathway is also assessed. KEGG analysis indicated that 65 DEGs related to apoptosis were identified as significant (Table 4). When the three experimental groups were compared with each other, *PTPN13* was notably up-regulated in both AD and AD_Pls groups. *ITPR2* was up-regulated by 5.06-folds in the AD group, whereas only 2.92-folds in the AD_Pls group. When the AD and AD_Pls groups were compared, the expression levels of *DDIT3* were increased whereas *CASP2* level was decreased after treatment with Pls.

### Other Differentially Expressed Genes

Besides the aforementioned DEGs, the transcription level of *BHLHB9* was up-regulated thousands of times in both AD and AD_Pls groups, whereas *KLHL11*, *AKAP2*, DGKK, *ZNF445*, *PDE1A*, and *MYH15* mRNAs were up-regulated dozens of times (Table 5). On the contrary, the transcription levels of *GPCR22* and *TRAPPC* were significantly down-regulated in both AD and AD_Pls groups. Finally, when the AD and AD_Pls groups were compared, the expression levels of *MATR3*, *CELF6*, and *ADORA2* in AD_Pls cells were significantly down-regulated after treatment with Pls (Table 5).

**TABLE 5:** Other DEGs identified among different groups.

Gene name	Fold change (AD model/control)	Fold change (AD model + Pls/control)	Description
*BHLHB9*	1347.47	7905.61	Basic helix-loop-helix family member b9
*PDE1A*	27.51	16.27	Phosphodiesterase 1A
*ARMCX4*	17.29	7.53	Armadillo repeat containing X-linked 4
*TIAF1*	16.03	2.03	TGFB1-induced anti-apoptotic factor 1
*CELF6*	0.77	0.03	CUGBP Elav-like family member 6
*ADORA2A*	0.92	0.05	Adenosine A2a receptor
*MATR3*	5.09	0.03	Matrin 3

### Quantitative RT-PCR

**FIGURE 3:**
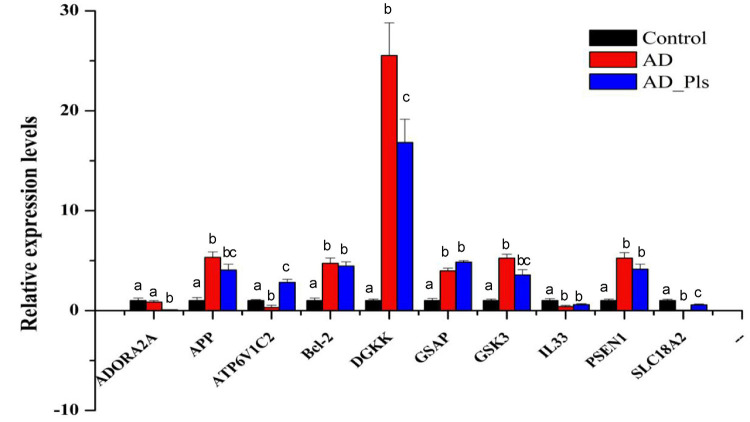
A comparison of the expression levels of several genes in AD model, AD model + Pls, and control groups. These genes were selected as KEGG analysis indicated that they were AD-related (*APP, GSAP, GSK3*, and *PSEN1*), autophagy-related (*Bcl-2*), fatty acid metabolism-related (*DGKK*), or their mRNAs levels were significantly changed among different groups (*ATP6V1C2, SLC18A2*, and *IL33*). The expression level of each gene in the control group was set as 1, and that in AD and AD_Pls groups was quantified relative to it. *GAPDH* was selected as an endogenous control. The results were represented as average ± SD (*n* = 3), and different letters denoted a significant difference between each group at *p* < 0.05 (Duncan’s new multiple range test).

To validate the transcriptome results, 10 transcripts were selected and further analyzed by qRT-PCR. As expected, the expression patterns of *ADORA2A*, *APP*, *ATP6V1C2*, *Bcl-2*, *DGKK*, *GSAP*, *GSK3*, *IL33*, *PSEN1*, and *SLC18A2* mRNAs were consistent with those obtained by RNA-seq (Figure 3). Compared with control cells, the expression levels of *DGKK* were markedly increased in AD and AD_Pls groups. *SLC18A2* and *ADORA2A* were the most significantly down-regulated transcripts in the AD group and AD_Pls groups, respectively. However, discrepancies were also observed between data obtained from the qRT-PCR and RNA-seq. For example, the up-regulation levels of *DGKK* mRNA in AD and AD_Pls groups obtained from qRT-PCR were 25.53 and 16.82, whereas these values were 30.68 and 27.51 when detected by RNA-seq. These discrepancies might result from different sensitivities among different technologies.

## Discussion

A key limitation in the field of neuroscience is the lack of suitable *in vitro* models resembling mature neurons. Although animal models are informative, they cannot provide a full explanation of the molecular mechanisms underlying neuronal function as human transcriptional networks are complex, with species-specific gene expression modulation patterns (Goldie et al., 2014). SH-SY5Y neuroblastoma cells have been used extensively to model networks and pathways related to human cognitive disorders. Although there is debate about the need to differentiate SH-SY5Y, an increasing number of studies have suggested the consideration of experimental methodology and applicability of the cell model in answering complex functional questions related to human neural architecture (Goldie et al., 2014). Previous studies have reported that the ATRA differentiation-BDNF maturation program not only induced significant increases in the expression of neuron-specific marker genes (such as *Sv2*, *NeuN*, and *NPY*), but also yielded SH-SY5Y cells with phenotype approaching mature neurons (Agholme et al., 2010; Goldie et al., 2014). Consistent with these studies, the expression levels of *Sv2C* in AD model and AD_Pls cells were 3.88- and 3.36-folds higher than that of the controls, and the *NPY2R* levels were up-regulated by 8.78- and 6.98-folds in AD and AD_Pls groups when compared with the controls (Supplementary Table 2). These data indicated that differentiated SH-SY5Y cells displayed mature neuron-like appearance and function. These neuron-like cells were further treated to obtain AD models, and subsequently used to investigate the potential role of seafood-derived Pls in AD pathogenesis.

Down-regulation of OXPHOS genes in animal models of AD has been reported in previous studies, and it is suggested that these reductions could trigger an overall bioenergetic crisis in the neurons, resulting in cell death (Area-Gomez and Schon, 2017; Mastroeni et al., 2017). Altered Ca^2+^ homeostasis in AD and AD_Pls cells was also observed at the transcriptional level in this study, which has been considered as an upstream event of AD pathogenesis and often occurs before the development of overt symptoms (Tong et al., 2018). Collectively, these data indicate that exposure to Aβ_1–42_ cause energy defects and Ca^2+^ dysregulation in SH-SY5Y cells, whereas PLs might effectively attenuate this crisis due to less significant down-regulation of these genes.

The PI3K-AKT/mTOR signaling pathway has been considered as the root cause of neuropsychiatric disorders such as AD (Sharma and Mehan, 2021). Up-regulation of PI3K-AKT/mTOR signaling pathway is associated with axonal dysregulation, leading to harmful consequences including over-production of reactive oxygen species, mitochondrial instability, lower oxidative phosphorylation and ATP levels, and even neuronal apoptosis (Seitz et al., 2012). Conversely, PI3K-AKT/mTOR signaling pathway inhibitors play a neuroprotective role, because of their effects in inhibiting cell apoptosis (Seitz et al., 2012). Consistent with those previous studies, Table 3 shows that the up-regulation degrees of DEGs related to PI3K-AKT/mTOR and MAPK signaling pathways were less significant after Pls treatment. These data indicate the PI3K-AKT/mTOR and MAPK signaling pathway is activated after Aβ_1–42_ exposure, whereas Pls treatment inhibits the activation of these signaling pathways.

*ATP6V1C2* encodes vacuolar-H^+^ ATPase (V-ATPase), which is a proton pump that is required for acidification of lysosome/vacuole (Zhao et al., 2018). Thus, V-ATPase would influence cellular processes such as endocytosis, vacuole fusion and protein degradation in eukaryotes. As lysosomes are the final-degradation organelles of Aβ peptide and acidic environment is the determinant of hydrolytic enzyme activation, it is speculated that down-regulation of *ATP6V1C2* mRNA levels in AD model cells would lead to increasing of pH value in lysosomes, resulting in inactivation of peptidases and inefficient Aβ clearance (Zhao et al., 2018; Zhou et al., 2021). However, the acidic lysosomal environment recovered after Pls induction by elevated *ATP6V1C2* transcript levels in AD_Pls cells (Table 3). In addition, *ATP6V1C2* also modulates the concentration of neurotransmitters into synaptic vesicles and is crucial in synaptic transmission (Zhao et al., 2018). A very recent study has reported that the pleiotropic roles of low *ATP6V1A* levels in AD pathogenesis are mediated via the synaptic vesicle cycle, phagosome, and oxidative phosphorylation (Zhou et al., 2021), which is consistent with results of this study.

*SLC18A2* is another notable DEG involved in synaptic vesicle cycle, which encodes a neurotransmitter transporter responsible for the packaging of small molecule neurotransmitters (acetylcholine, histamine, dopamine, norepinephrine, epinephrine, and serotonin) into synaptic vesicles (Hu et al., 2020). *SLC18A2* is expressed in monoaminergic neurons of the central nervous system, and abnormal expression of *SLC18A2* has been proposed to contribute to vulnerability toward epilepsy-related psychiatric disorders and cognitive impairment in adults (Markos et al., 2016; Treble-Barna et al., 2017). The *SLC18A2*

inhibitor (Deutetrabenazine) is proved to be effective in the cure of involuntary movements in patients with tardive dyskinesia, and the *SLC18A2* blocker (Tetrabenazine) is the only US Food and Drug Administration-approved drug for Huntington’s disease (Hu et al., 2020). In this study, the transcript levels of *ATP6V1C2* and *SLC18A2* were significantly down-regulated in AD model, yet their levels were quickly recovered after further treatment with Pls (Table 4). Thus, it is suspected that Pls may also alleviate Aβ_1–42_-induced neurotoxicity through modulating neurotransmitter system.

Autophagy plays a modulatory role in the internalization and uptake of Aβ, and likely impacts the degradation or formation of Aβ plaques (Bordi et al., 2016). Nevertheless, sometimes conflicting results were obtained according to earlier investigations of autophagy induction in AD models (Bordi et al., 2016). Through a custom-designed microarray analysis, a previous study reported that genes related to autophagosome formation and lysosomal biogenesis were up-regulated, whereas the autolysosomal proteolytic function was not evidently altered at early stages of AD (Lipinski et al., 2010). Results of this study were consistent with these reports, as shown by the overall up-regulation of autophagy Initiation, autophagophore formation and elongation, and autophagosome-lysosome fusion-related genes, whereas the expression levels of lysosomal proteolysis were not evidently changed. Therefore, it is hypothesized that the activation of autophagy in AD model cells may represent an acute attempt by the affected neuronal cells to rid themselves of the harmful effects of Aβ_1–42_ exposure. However, abnormally accelerated endocytosis and accumulation of Aβ_1–42_ eventually become counterproductive as lysosomal proteolysis function is insufficient, leading to the acceleration of AD onset.

Among other DEGs, the up-regulation of *BHLHB9* was most notable (Table 5). BHLHB9 is also known as GPCR-associated sorting protein 3 (GASP-3), it promotes neurosynaptogenesis by influencing the phosphorylation and proteolytic processing of APP and PSENs in transgenic mice (Mishra and Heese, 2011). The marked up-regulation of *BHLHB9* mRNA levels in both the AD and AD_Pls groups may be related to the complex neural network and synaptic structures formed in AD and AD_Pls cells compared with control cells. However, it is noticed the *BHLHB9* mRNA level is 5.87-fold higher after further treatment with Pls. This result is consistent with the identified roles of *BHLHB9* in improving memory and learning abilities in animal models, as well as the expected effect of Pls on alleviating AD pathology (Mishra and Heese, 2011). Other DEGs in Table 5 are also relevant to neurodegenerative diseases. *PDEs* are targets for therapy of AD, as many PDE inhibitors have shown encouraging cognitive improvement effects (Nabavi et al., 2019). *ARMCX4*, also known as *GASP-4*, is one of the highly dysregulated priority genes in brains of Parkinson’s disease (Abu-Helo and Simonin, 2010). *TIAF1* encodes a small TGF-β1-induced factor, and a significant up-regulation of Aβ levels occurred rapidly following TIAF1 self-association in degenerating and dead neurons (Chang, 2009).

Table 5 also showed that expression levels of *ADORA2*, *CELF6*, and *MATR3* were significantly altered after Pls treatment. *MATR3* is one of the newly identified dementia-causing genes (Park et al., 2020). *ADORA2A* encodes the adenosine A2A receptor, which is a GPCR that mediates synaptic transmission and neuronal excitability in the central nervous system (Domenici et al., 2019). Moreover, *ADORA2A* has been reportedly essential for Aβ_1–42_ toxicity as Aβ_1–42_ did not induce learning deficits or synaptotoxicity in *ADORA2A* knockout mice (Chen et al., 2007). In addition, preclinical data have also supported the use of adenosine A2A receptor as therapeutic target in neuropsychiatric disorders (Domenici et al., 2019). *CELF6* encodes a RNA-binding protein, which is highly expressed in several monoaminergic cell populations and in cells of the hypothalamus commonly targeted in psychiatry. A recent study has revealed that many targets mRNA of *CELF6* encode proteins involved in synaptic transmission (Rieger et al., 2020). When the transcriptional alterations of *ATP6V1C2, SLC18A2*, *ADORA2*, and *CELF6* are considered together, it is speculated that seafood-derived Pls alleviate the pathology of AD mainly by modulating synaptic vesicle trafficking, promoting neurotransmitter transport, and synaptic transmission.

The expression of several mRNAs encoding GPCRs (*ADORA2A*) and GASPs (*BHLHB9* and *ARMCX4*) were significantly altered in this study. These findings were consistent with previous research, as it has been reported that Pls activate orphan GPCRs to enhance the phosphorylation of ERK and Akt kinases, resulting in the inhibition of caspase-3 activity and thus inhibiting neuronal cell death (Hossain et al., 2016). Besides this, GPCRs also regulate tau phosphorylation and Ca^2+^ dysregulation through various cellular kinases in AD neurons (Chidambaram and Chinnathambi, 2020). Based on these reports and findings of this study, it is suspected that GPCRs are extensively involved in AD pathogenesis, and they may contribute to the protective role of seafood-derived Pls in SH-SY5Y neuronal-like cells.

It is worth noting that although the transcription levels of many genes related to AD pathogenesis, autophagy, endocytosis, synaptic vesicle trafficking, and apoptosis were significantly altered among different groups, obvious morphology differences were not observed among these neuron-like cells after further treatment with Pls or Aβ_1–42_. These results may be related to the short treatment time (24 h) applied in this study. Therefore, longer treatment time will be used in the next study, to further validate these transcriptional alterations by morphological changes. In addition, the protect effective of sea-food derived Pls needs to be further confirmed at the protein level.

## Conclusion

In summary, the alteration of transcriptional profiles in AD model and AD_Pls cells were investigated, the relevance of PI3K-Akt/mTOR and MAPK signaling pathway, autophagy, endocytosis, synaptic vesicle trafficking, autophagy and apoptosis to the pathogenesis of AD, as well as the potential role of seafood-derived Pls in relieving AD progression were analyzed. The obtained data confirmed that the activation of autophagy in AD model cells is the first response to Aβ_1–42_ impairment, while ATP depletion, deficient lysosomal proteolytic function are the second step. DEGs significantly identified among different groups suggested that therapeutic roles of seafood-derived Pls are mediated through accelerating the toxic Aβ_1–42_ clearance, promoting neurotransmitter transport and synaptic transmission, and facilitating the formation of complex neural network and synaptic structures in AD model SH-SY5Y neuronal-like cells. Results of this work also provided evidence that the GPCRs implicate in Pls-signaling in neuronal cells. However, further *in vivo* studies are needed to validate the effect of Pls on AD proposed in this study at protein level, and to assess the potential use of seafood-derived Pls as an effective therapeutic agent for AD.

## Data Availability Statement

The datasets presented in this study can be found in online repositories. The names of the repository/repositories and accession number(s) can be found below: NCBI under accession PRJNA728528.

## Author Contributions

JF and GS: study concept and design and participated in the drafting of the article. JF and XC: acquisition of data. QW and SG: analysis of samples and data interpretation. QS and MZ: critical revision of the manuscript for important intellectual content, and study supervision. All authors contributed to the article and approved the submitted version.

## Conflict of Interest

The authors declare that the research was conducted in the absence of any commercial or financial relationships that could be construed as a potential conflict of interest.

## Publisher’s Note

All claims expressed in this article are solely those of the authors and do not necessarily represent those of their affiliated organizations, or those of the publisher, the editors and the reviewers. Any product that may be evaluated in this article, or claim that may be made by its manufacturer, is not guaranteed or endorsed by the publisher.
