# Supplementary figures and images for: Protect Effects of Seafood-Derived Plasmalogens Against Amyloid-Beta (1–42) Induced Toxicity via Modulating the Transcripts Related to Endocytosis, Autophagy, Apoptosis, Neurotransmitter Release and Synaptic Transmission in SH-SY5Y Cells
Source: Front Aging Neurosci. 2021 Nov 26;13:773713. doi: 10.3389/fnagi.2021.773713 (PMC8662987; doi:10.3389/fnagi.2021.773713)

**Supplementary Fig. S1. Pearson correlations between nine samples belong to three groups.**

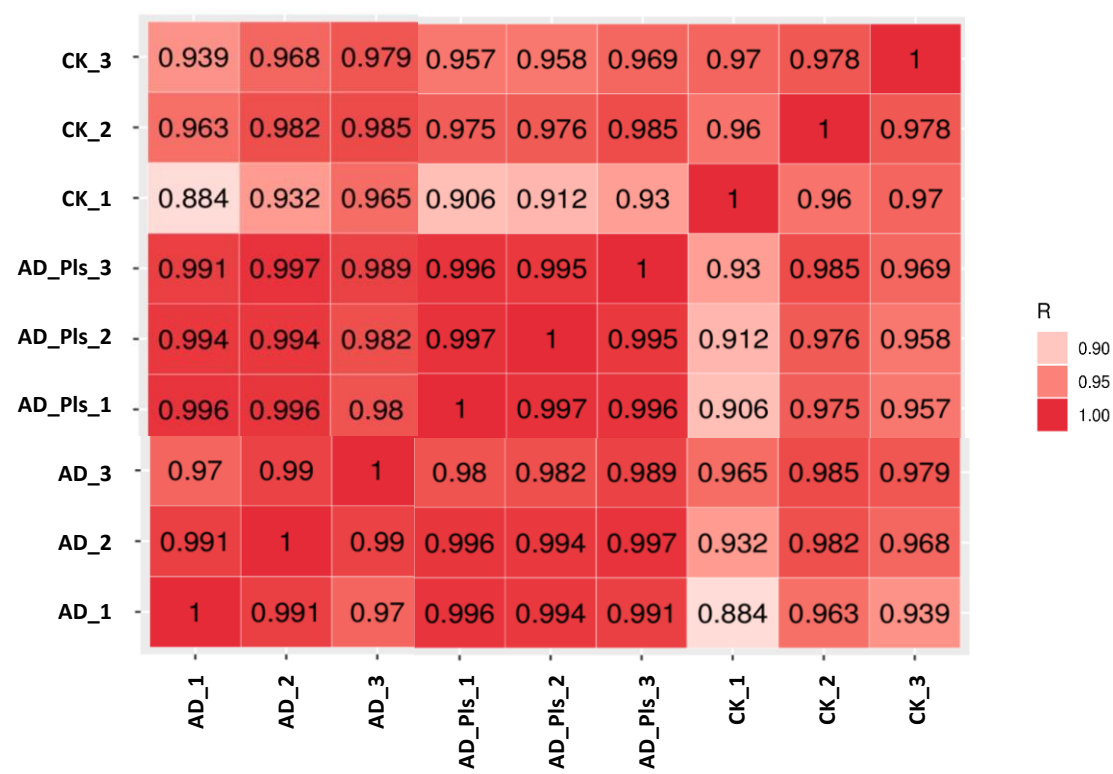

**Fig. S1**

Supplement: Supplementary file 1 [file Image_1.pdf]
