# Supplementary material for: Protect Effects of Seafood-Derived Plasmalogens Against Amyloid-Beta (1–42) Induced Toxicity via Modulating the Transcripts Related to Endocytosis, Autophagy, Apoptosis, Neurotransmitter Release and Synaptic Transmission in SH-SY5Y Cells
Source: Front Aging Neurosci. 2021 Nov 26;13:773713. doi: 10.3389/fnagi.2021.773713 (PMC8662987; doi:10.3389/fnagi.2021.773713)
Supplement: Supplementary file 2 [file Table_1.DOC]

**Table S1.** Sequences of the Primers Used in the Quantitative RT-PCR

| **Gene Name** | **Forward Primers (5´-3´)** | **Reverse Primers (5´-3´)** |
| --- | --- | --- |
| *ADORA2A* | TGTGGCTCAACAGCAACCTG | CGTGAGGACCAGGACGAAGC |
| *APP* | CATCATGGTGTGGTGGAG | GCGATAATGAGTAAATCATAAAAC |
| *ATP6V1C2* | GAAGGCCAACCTGGAGAACT | AAGCTTGACTTGGGGACGAT |
| *Bcl-2* | CGGGAGAACAGGGTATGATA | CCACCGAACTCAAAGAAGG |
| *DGKK* | GGGTGGGAGCCTCAAACAAT | TGGTCAGAGGTCTCGTGTCT |
| *GSAP* | GTTTCAGGTTGGTACACGTT | TGCCACTGTATGCCTTC |
| *GSK3* | CCTGGCCCCCGGGTGTAAATAGAT | GGGTGGGGTGAGGAGGGAGTAGAC |
| *IL33* | GTGACGGTGTTGATGGTAAGAT | AGCTCCACAGAGTGTTCCTTG |
| *PARL* | AAGTGCTTTGATTCCTCCTG | GCCTGTAAACCCAACAGTAAA |
| *PSEN1* | GGTGGCTGTTTTATGTCCCAA | CAACCACACCATTGTTGAGGA |
| *SLC18A2* | CAGACAGCTTCCAGAGCATCTT | AAGTGTCAGGTCTCTGGTAGCATT |
| *GAPDH* | TTGGTATCGTGGAAGGACTC | ACAGTCTTCTGGGTGGCAGT |
